# Experimental Evaluation of 79 and 300 GHz Radar Performance in Fire Environments †

**DOI:** 10.3390/s21020439

**Published:** 2021-01-09

**Authors:** Aleksandr Bystrov, Liam Daniel, Edward Hoare, Fatemeh Norouzian, Mikhail Cherniakov, Marina Gashinova

**Affiliations:** School of Engineering, University of Birmingham, Birmingham B15 2TT, UK; l.y.daniel@bham.ac.uk (L.D.); e.g.hoare@bham.ac.uk (E.H.); f.norouzian@bham.ac.uk (F.N.); m.cherniakov@bham.ac.uk (M.C.); m.s.gashinova@bham.ac.uk (M.G.)

**Keywords:** radar imaging, millimetre wave propagation, electromagnetic wave absorption, fire safety

## Abstract

This paper presents an experimental study of the propagation of mm-wave/low-THz signals in the frequency ranges of 79 and 300 GHz through fire. Radar performance was investigated in various real scenarios, including fire with strong flame, dense smoke and water vapour. A stereo video camera and a LIDAR were used as a comparison with other common types of sensors. The ability of radars to enable the visibility of objects in fire environments was proven. In all scenarios, the radar signal attenuation was measured, and in the case of steam was compared with theoretical calculations. The analysis of the experimental results allows us to conclude that there are good prospects for millimetre wave and Low Terahertz radar in the field of firefighting imaging equipment.

## 1. Introduction

Extinguishing fires in buildings is a dangerous job where conditions due to flame, smoke and steam can change very quickly for the worse, partially, or fully, obscuring visibility. Therefore, an important task is the development of sensors able to see through such obscurants to locate dangerous spots, map the scene and detect vulnerable actors.

Our previous studies have shown the advantage of Low Terahertz (Low THz) radar over optical sensors including LIDAR, to enable high resolution imagery in artificially generated fog [1]. Similar conclusions for millimetre wave radar in the case of fire were made in a review [2]. In [3,4], the practical feasibility of implementing a 76 GHz radar to obtain information about objects in a fire was shown. At the same time, as can be seen from [2], the use of radars operating in the frequency range above 100 GHz has not been sufficiently studied.

Increasing the operational frequency offers several advantages including reducing antenna aperture for the same beamwidth, with a corresponding reduction in size and mass, which is a significant advantage for portable applications in such extreme physical environments. Importantly, an increase in operational frequency offers the potential to significantly increase the transmitted bandwidth to provide very high range resolution, in the order of centimetres, to provide an overall resolution capable of producing useful radar images. Modern millimetre-wave radar techniques and new sensing approaches allow us to determine the parameters of objects with high accuracy [5,6,7].

Additionally, as the operational frequency increases, the wavelength decreases, which provides increased interaction with the physical surface texture of the target objects, giving additional information due to more diffuse scattering.

To provide a useful instrument for use in fire applications, the radar should be capable of producing an output to the operator similar to a video image; this relies on high range and azimuth resolution. For an imaging application, the combination of high range resolution, high angular resolution and scattering effects due to the increased sensitivity to texture all provide the potential to produce images similar to IR sensors, without the obvious disadvantage of using IR sensing in a fire environment.

Technology is coming closer to developing Low THz radar components. Silicon Germanium technology is advancing to the point where 120 and 300 GHz transceiver products are now readily available on the market. For example, Silicon Radar GmbH produces a complete 120 GHz radar monolithic microwave integrated circuit (MMIC) front end with integrated antennas in an 8 × 8 mm^2^ package [8]. The 300 GHz prototypes are currently available in the same very small package [8]. Such developments offer the prospect of large-scale production at very low cost in the near future.

However, signal propagation issues in a real fire environment are fundamentally unknown at these frequencies. In this paper, we present an experimental study of the propagation of electromagnetic signals through fire in the frequency ranges of 79 and 300 GHz. The 79 GHz radar frequency corresponds to the typical frequency range of automotive radars. The 300 GHz radar frequency is of particular interest as an example of a Low-THz radar. We compare the performance of radar, stereo video camera and LIDAR in various fire-related obscurants, ranging from strong open flames, dense smoke and water vapour. This paper is an extended presentation of the results of our study compared to those published in [9,10]. Compared to previous works, in this extended paper, we examined in more detail the physical properties of the flame, compared the performance of the radar with the LIDAR, presented the results of the measured and calculated signal attenuation at different temperatures, investigated the signal range profile change over time, and refined other calculations and graphs. The measurements were taken at a training facility at Oldbury Fire Station, West Midlands, UK, under the supervision of professional firefighters.

This paper is structured as follows. In Section 2, we define the physical environment of fire and discuss the challenges of obtaining usable imagery under such conditions. We also discuss the methods used to characterize the propagation channel through fire obscurant components. In Section 3, the organization of the experimental work is described, and the parameters of the measurement systems are presented. In Section 4, the experimental results for various fire scenarios are presented and discussed, and a comparison of 79 and 300 GHz radars with a stereo video camera and a LIDAR is made. The results of measuring the attenuation of radar signals through fire are shown in Section 5; in the case of water vapour, they are compared with theoretical calculation. Finally, in Section 6, conclusions are presented together with future research plans.

## 2. Microwave Propagation in Fire

To understand the mechanisms of radar signal propagation through fire, it is necessary first to define what the actual physical content behind the general term “fire” is. As shown in [11,12,13,14], fire is composed of many different substances. For the most part, fire is a mixture of hot gases; the reaction also produces carbon dioxide, water vapour, light and heat. If combustion is incomplete, a fire may also give off tiny solid particles of soot or ash, as well as additional gases, such as carbon monoxide or sulphur dioxide.

If the flame is hot enough, the gases are ionized, and flame acquires the well-known properties of an electric plasma [15]. A plasma is an ionized gas that is reflective to electromagnetic waves. In a flame, the amount of ionization depends on the temperature. Typical compartment fire temperatures are about 400–600 °C, and under usual conditions, do not exceed 1000 °C [16]. As concluded in [15], temperatures from everyday flames due to the burning of wood, charcoal, gasoline, propane or natural gas, or even higher temperatures, are typically not hot enough to generate a significant plasma. However, there is a small amount of ionization that happens in an ordinary fire, and in practice, fire may be defined as a partially ionized plasma [17]. Due to the presence of charged particles, flames may be considered to be conductive [18].

The fire components discussed above affect the propagation of electromagnetic waves in various ways [19,20,21]. Water vapour and oxygen cause strong attenuation of electromagnetic waves at certain frequencies [22] due to absorption. In a fire environment, particle scatter occurs from carbon particles swept up into the combustion region by convection currents. The absorption of radar signals will depend on the properties of smoke and soot particles and their size relative to the signal wavelength [23,24]. Smoke particles generated by combustion are small compared to radar signal wavelengths; therefore, scattering obeys the Rayleigh approximation, and as the frequency increases, the scattering increases, too.

Ionized electrons in a plasma interact with electromagnetic waves, resulting in dispersion and attenuation. It was shown in [25] that as frequency increases the transmittance through a plasma increases and the attenuation decreases. Authors in [26] conclude that the problem of blocking electromagnetic signals in plasma can be effectively solved by moving the incident wave frequency to the THz wave frequency band.

There are theoretical methods [22,24] that allow us to consider the influence of each factor of fire separately, if their parameters are known (particle composition and size, vapour density, temperature, etc.). However, it is not known how the influence of these factors manifests itself and combines in a real fire. Different combustion conditions (the type of combustible material—access to oxygen, presence of water, combustion temperature, etc.) produce different types of flame and smoke. Therefore, to answer the question about the performance of radar in fire environments, it is necessary to conduct experiments in conditions as close as possible to real fire scenarios.

## 3. The Experiment Setup

In order to analyse the performance of 79 and 300 GHz radar in different fire environments, we investigated three scenarios that occur during a fire, namely, dense flame, dense smoke, as well as a mixture of steam and particulates, which typically occurs when fire is being extinguished. In the experiments, the fire area was scanned using various sensors. The images obtained were compared with reference images in the absence of fire.

**Figure 1:**
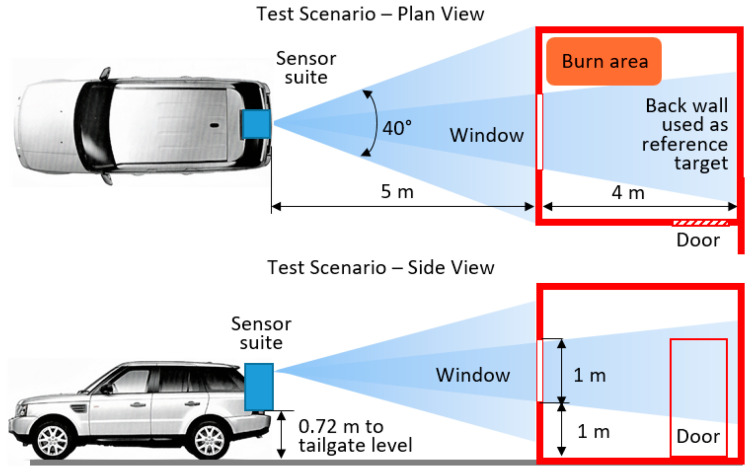
Test scenario.

A schematic of the experimental setup is shown in Figure 1. The fire was ignited in a 4 × 4 m^2^ room, where an unglazed 1 × 1 m^2^ window was installed at the front to enable viewing of the interior. The room was in a concrete building specially designed for training firefighters. The window was equipped with steel shutters.

**Figure 2:**
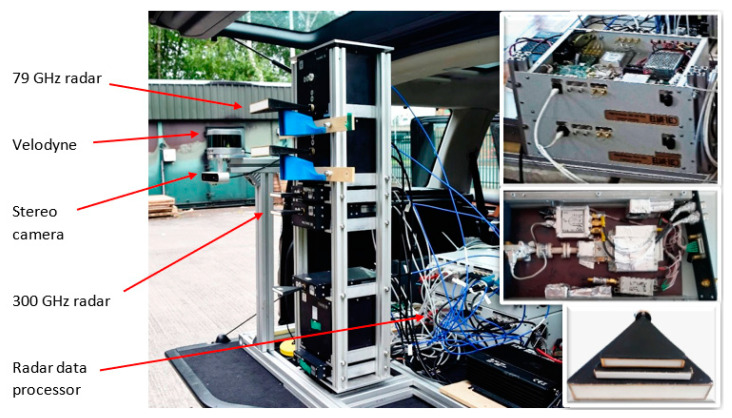
Sensor suite. The insets show (from top to bottom): radar data processor, radar board, antennas.

In addition to radars, the fire area was instrumented with a LIDAR and a stereo video camera. The distance from the vehicle mounted sensors to the window was 5 m. The image resolution of ZED Stereo Camera (Stereolabs, San Francisco, CA, USA) was set to 1280 × 720 pixels, and frame rate was 30 fps. The LIDAR was a Velodyne 32 (Velodyne Lidar, San Jose, CA, USA) LIDAR with 32 vertically aligned beams allowing a ±15° elevation field of view with 300 rpm frame rate. A photo of the sensors mounted in the rear of the vehicle is shown in Figure 2.

The antenna parameters and parameters of the radars are shown in Table 1. The radars were mounted on a turntable and scanned ±20° field of view in azimuth with the step of 0.25° (Figure 1). The scanning frequency of the full scene was 0.83 Hz, defining the frame time of 1.2 s. The number of FMCW chirps per scan was 158. Each measurement consisted of 40 separate frames and lasted about 48 s. As can be seen from the table, the 300 GHz radar has lower transmit power than 79 GHz radar; however, since our goal is to investigate signal losses, the absolute power is of no consequence as long as the signal is above the noise/clutter.

## 4. The Results of Experiment

### 4.1. Background Imaging

In our first experiment, the back wall of the room was used as a reference surface. Radar scan data were recorded by a data processor and translated into images (radar maps). The azimuthal resolution is equal to the antenna azimuth beamwidth θ, multiplied by the range R, and the range resolution ΔR is presented in Table 1. As the antenna scans, cells of size θR × ΔR are generated to form a map. The size of cells varies with range. Because the azimuthal step Δθ = 0.25° is lower than the beamwidth θ = 2.2°, cells are partially overlapped. The size (range × azimuth) of the 79 GHz radar map was 318 × 160 and the size of the 300 GHz radar map was 1137 × 160.

**Figure 3:**
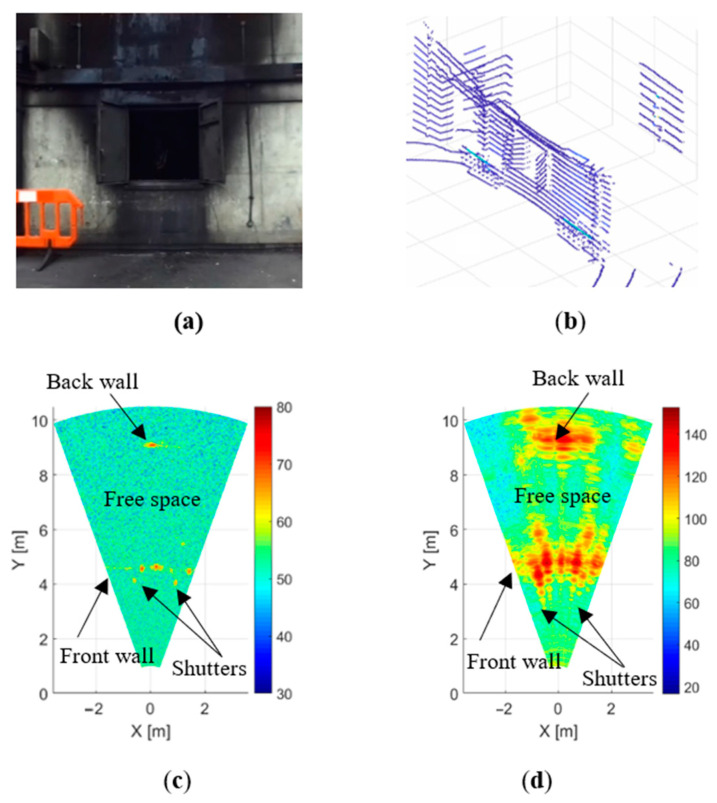
Measurement of empty scene with no flame, smoke or steam: (**a**) camera, (**b**) LIDAR, (**c**) 300 GHz radar and (**d**) 79 GHz radar.

Radar maps of the scene with different elements of fire were compared with the background map of an empty scene in the absence of fire. In Figure 3, the background images of the scene (optical, LIDAR point cloud and radar 2D maps) are shown to define the reference map. The colour bar in this and in the following figures indicates the signal power in dB on an arbitrary scale.

In the absence of lighting inside the room, the back wall is not well lit and can hardly been seen in the camera image (Figure 3a), while the Velodyne LIDAR provides a very clear three-dimensional point-cloud map of the scene (Figure 3b). Window shutters are clearly distinguishable as well as the door on the left, plastic fences and a back wall.

In the images obtained by the radars (Figure 3c,d), the front wall and the rear wall of the room are clearly visible through the open window. The power of the reflected signal at a frequency of 79 GHz (Figure 3d) was high, and we can clearly see the reflection from the front wall of the room and from the back wall. The open metal window shutters produce strong reflections and are visible on either side of the window. Although the power of the 300 GHz radar was lower (Figure 3c), we can see reflections from the front and back walls of the room as well as from the shutters. The width of the reflected signal peaks for a 300 GHz radar is significantly narrower than that of a 79 GHz radar, which is determined by the lower output power of this radar and, accordingly, the worse SNR.

The radars allow measurement of the distance to the objects of interest with sufficient accuracy (see Table 1). At the same time, they do not provide a detailed image comparable to a camera video image due to lower resolution in range and azimuth. Because the goal of our experiment is to measure signal attenuation in a fire, the radar image resolution is not a significant factor.

### 4.2. Dense Flame Environment

The second experiment was conducted in a high flame environment with little smoke. To create the conditions arising from such a fire, wooden pallets were ignited with the windows closed. The fire then went into the growth stage and spread rapidly through the compartment, burning from floor to ceiling. After the fire was fully developed, the metal window shutters were opened. The temperature inside the testing compartment was about 600 °C with only slight deviations during the experiment.

**Figure 4:**
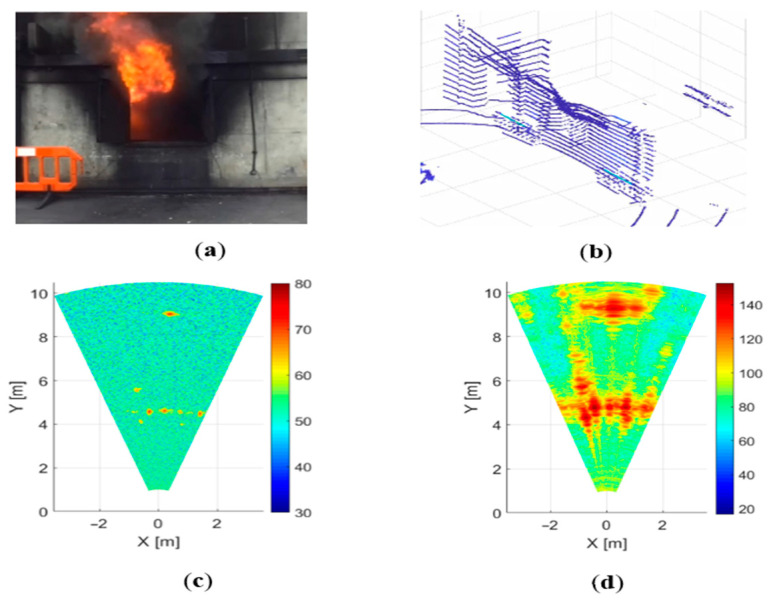
Dense flame environment: (**a**) camera, (**b**) LIDAR, (**c**) 300 GHz radar and (**d**) 79 GHz radar.

The camera image is presented in Figure 4a. The image shows only flame and smoke pouring out of the window; objects in the room are not visible. As can be seen from Figure 4b, the LIDAR can see the back wall of the room only partially. The effect of the flame on the radar images is almost imperceptible, at least visually. This is clearly seen from a comparison of Figure 3b and Figure 4b, or Figure 3c and Figure 4c, where the window details and the back wall of the room are clearly visible.

### 4.3. Dense Smoke Environment

**Figure 5:**
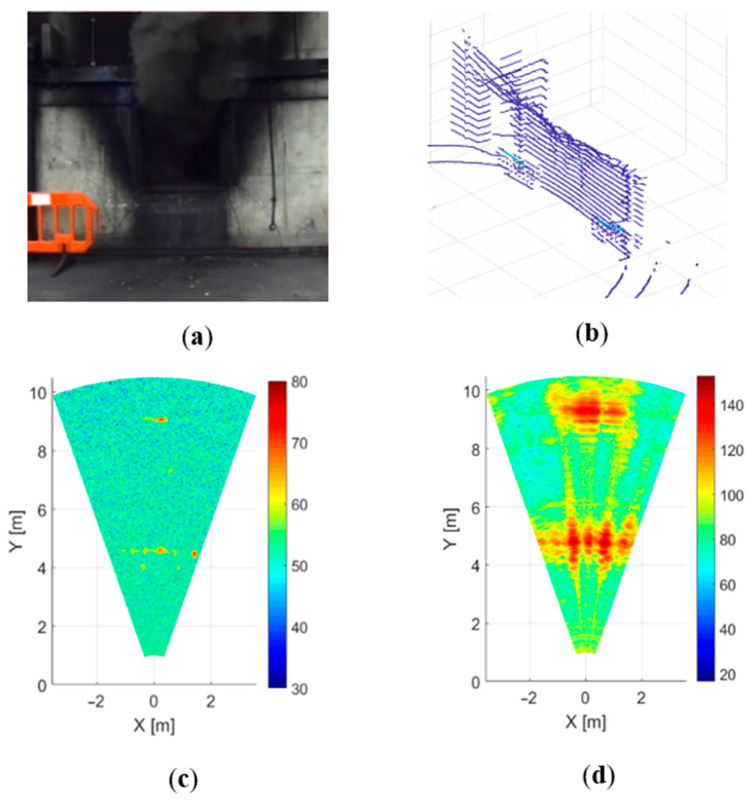
Dense smoke environment:(**a**) camera, (**b**) LIDAR, (**c**) 300 GHz radar and (**d**) 79 GHz radar.

Burning newspapers and cardboard, in addition to wood-based materials, were used to create a smoke fire environment. The temperature inside the testing compartment was about 400 °C, which is lower than the previous test. During this measurement, the smoke intensity remained visually unchanged. Unlike the previous experiment, smoke is a more serious problem for LIDAR, and the back wall is not visible in the Velodyne image (Figure 5b). The results obtained using radars, presented in Figure 5b,c, are generally similar to those shown in Figure 4b,c, and therefore, all conclusions made regarding the previous experiment can be fully applied to this scenario.

### 4.4. Steam and Particulate Mix Environment

**Figure 6:**
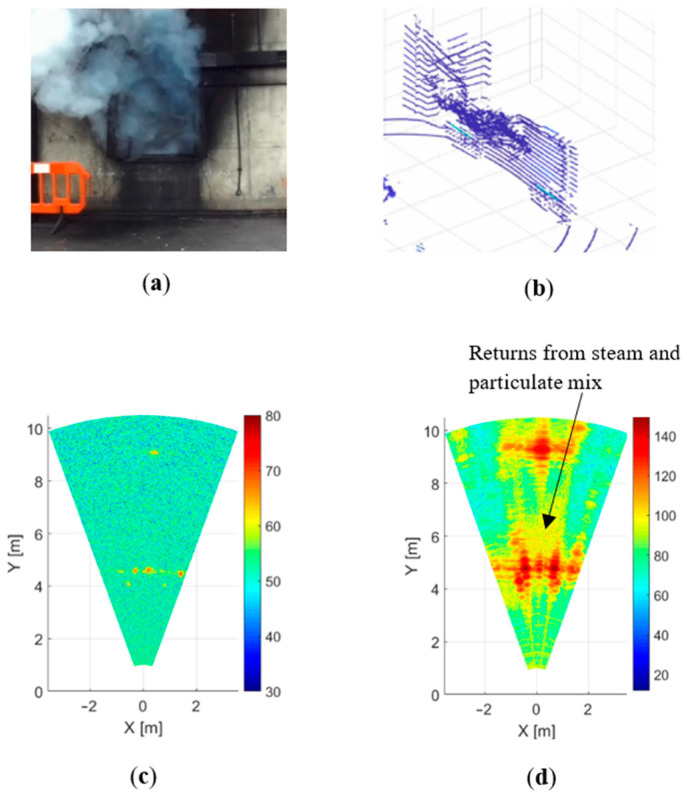
Steam and particulate mix environment: (**a**) camera, (**b**) LIDAR, (**c**) 300 GHz radar and (**d**) 79 GHz radar.

In the third experiment, some differences in the recorded images were observed in the case of a dense water vapour (steam) environment, shown in Figure 6. In our experiments, these conditions were created by extinguishing the source of fire with water from a fire hose. The room temperature in this experiment was about 230 °C. In the first seconds, the maximum concentration of steam was achieved, and over time the steam intensity decreased.

The camera and the LIDAR were unable to see through heavy steam (Figure 6a,b). In the case of the 300 GHz radar (Figure 6c), the power of the signal reflected from the back wall decreased significantly, but the wall was still visible. The radar with 79 GHz frequency clearly distinguishes the back wall of the room. In the image obtained from the 79 GHz radar, the reflection zones from steam and particulate mix are visible, although the intensity of such reflection is significantly lower than from the walls (Figure 6d). As can be seen from Figure 4, Figure 5 and Figure 6, in all the fire scenarios considered by us, the radar provides reliable image acquisition of objects under fire conditions.

In the next section, we will analyse 300 and 79 GHz radar signal propagation in various fire scenarios. The section will provide a numerical estimate of signal attenuation and in the case of steam, the experimental data will be compared with theoretical calculation.

## 5. Radar Signal Attenuation

We calculated signal attenuation by measuring the power of the signal reflected from the back wall compared with the reference signal in the absence of fire. The combustion conditions changed with time, but to a first approximation, we consider them constant during each experiment, which lasted approximately 48 s (see Section 2). Moreover, the combustion conditions were not the same throughout the room. In the ignition centre and in the centre of the room, the temperature and intensity of smoke and steam were higher; closer to the walls, they decreased to some extent. When analysing signal propagation, we did not consider this, so losses were averaged along this 4 m room.

**Figure 7:**
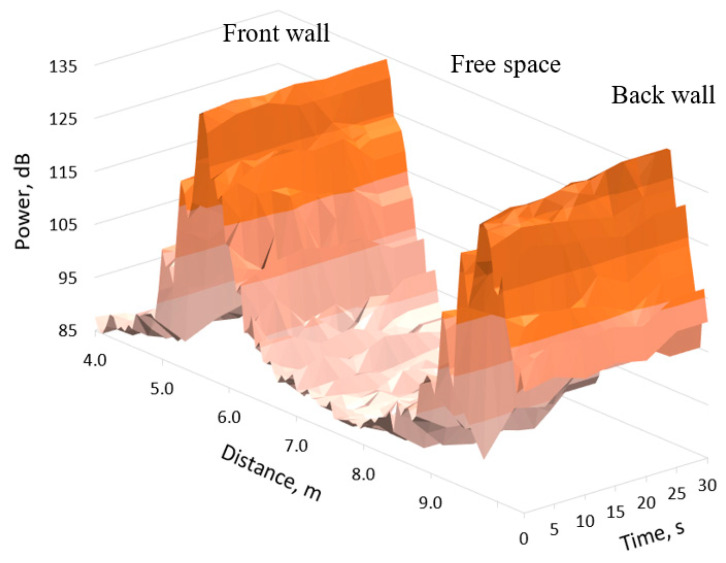
79 GHz radar signal range profile change over time in steam environment.

An averaged 79 GHz radar signal profile change over time during the first 30 s of experiment in the steam environment is shown in Figure 7. The power of the signal reflected from the back wall slightly increased over time as the steam cleared.

**Figure 8:**
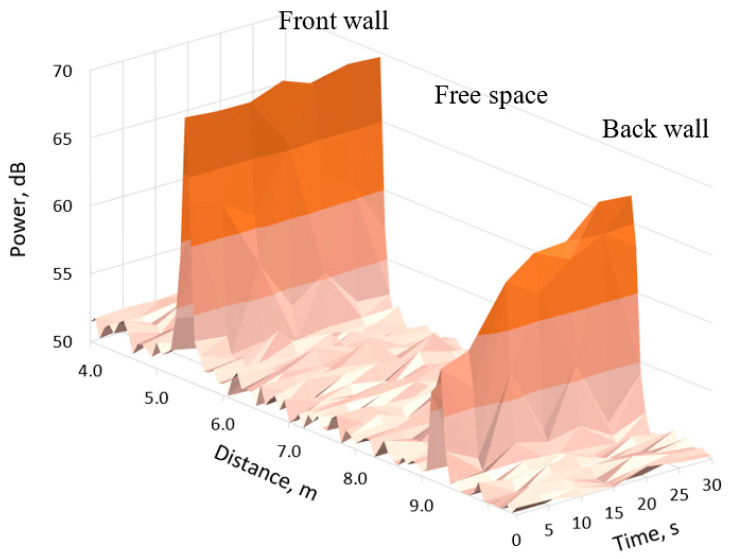
300 GHz radar signal range profile change over time in steam environment.

A similar result for a signal with a frequency of 300 GHz is presented in Figure 8. The width of the peaks of the signal reflected from the front and rear walls, in this case, is significantly narrower than in Figure 7, due to the wider signal bandwidth (see Table 1). Because of the lower power of the 300 GHz radar, we do not see significant reflection from the vapour and soot particulate mix. The power of the signal reflected from the back wall was significantly lower than the power of the signal reflected from the front wall, especially in the first 10–15 s. The comparison of Figure 7 and Figure 8 indicates the higher attenuation of the 300 GHz signal than of the 79 GHz signal.

The results of measured signal attenuation from different test scenarios are presented in Table 2. The results were averaged over the first 30 s of measurement from the start of each experiment. As can be seen from the table, flame and smoke did not significantly absorb 79 GHz radar signals. A signal with a frequency of 300 GHz was more susceptible to attenuation, although absorption in fire and smoke remained low.

The maximum attenuation of radar signals occurred in the steam and particulate mix environment. In this case, the average value was 0.88 dB/m for a signal with a frequency of 300 GHz and 0.45 dB/m for a signal with a frequency of 79 GHz. Nevertheless, even under these most unfavourable conditions, the radar made it possible to obtain an image of the back wall of the fire room.

**Figure 9:**
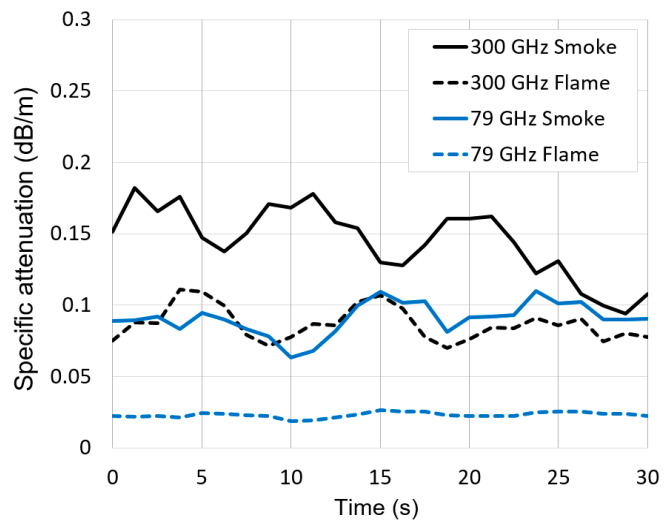
Radar signal measured specific attenuation in smoke and flame versus time.

The graphs of radar signal attenuation in smoke and flame as a function of time are presented in Figure 9. It can be seen from the graphs that the conditions of the experiment for the 30 s of measurement were stable and the signal attenuation did not change significantly (apart from a slight decrease in the attenuation of the 300 GHz signal in smoke in the last 5 s). The low signal absorption in flame probably indicates a weak plasma ionization; however, we cannot separate the attenuation in plasma from the absorption of signals resulting from combustion products. It can be assumed that the whole increase in signal attenuation in smoke compared to flame is caused by the influence of soot particles. Considering the limited number of experiments and low signal attenuation values, the results presented in the cases of strong flame and strong smoke should be regarded as indicative.

The measured specific attenuation in steam was compared with theoretical calculation. The observed attenuation is due to absorption and scattering by water vapour in steam. The scattering of particles strongly depends on the particle size and frequency of the incident wave. Three scattering regions based on dimensionless size parameter *k = 2πr/λ*, where *r* is the particle radius, are as follows [27]:

*k* << 1, Rayleigh scattering;
*k* ≈ 1, Mie scattering;
*k* >> 1, geometric scattering.

The particle sizes of steam are in the range of µm to mm [28]; therefore, particle scattering in steam obeys the Rayleigh approximation. The Rayleigh attenuation is independent of the particle size and is proportional to the steam density. The specific attenuation is calculated by [29]:

(1) $$A=\frac{0.819fM}{10^{3}\varepsilon^{\prime \prime }\left(1+\eta^{2}\right)}\left(dB/m\right),$$

where *f* is frequency in GHz, *M* is steam density in g⁄m^3^, *η = (2+ε′)/ε″*, *ε′* and *ε″* are the real and imaginary part of complex dielectric permittivity, which is obtained from the double-Debye model [30]. The steam density is function of temperature and obtained from [28]. The initial temperature was 230 °C, then the temperature gradually decreased, and 30 s after the start of the measurement, reduced to 134 °C. Steam density, corresponding to the temperature of saturated vapour, also decreased from *M* = 14,008 g/m^3^ to *M* = 1651 g/m^3^ [31].

**Figure 10:**
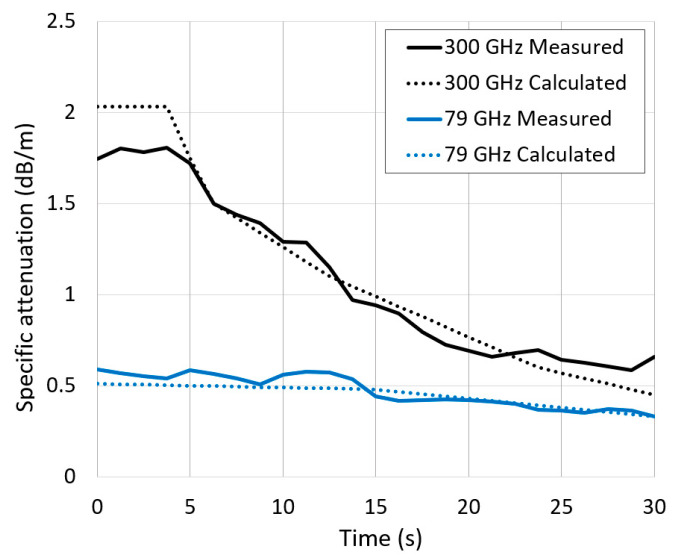
Radar signal measured and calculated specific attenuation in steam versus time.

The calculated 79 GHz signal attenuation using (1) for temperature of 230 °C was about 0.5 dB/m, which agrees with the average measured attenuation shown in Table 2. However, a reduction in attenuation was observed in the measured results over time as the steam cleared. This is shown in Figure 10 with a solid blue line. The calculated 79 GHz signal attenuation is also plotted by a dotted blue line using (1), considering temperature deviation as well as the change in density. The calculation and measurement results agree reasonably well, and they show a reduction in attenuation over time as water evaporates.

In the considered fire scenario, we observed a significant reduction in measured 300 GHz signal attenuation over time, from 1.75 to 0.6 dB/m. This is shown in Figure 10 with a solid black line. Given the fact that the travel distance of the signal, reflected from the back wall, was equal to 8 m, the received signal attenuation changed from 14 to 4.8 dB. The calculated 300 GHz signal attenuation is also plotted in Figure 10 by a dotted black line using (1). In general, there is good agreement between the measured and calculated signal attenuation.

**Figure 11:**
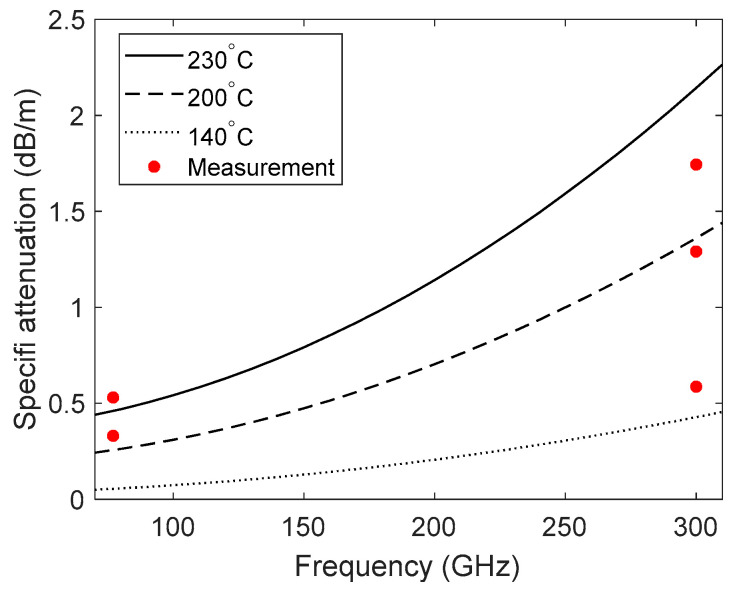
The calculated specific attenuation versus frequency in comparison with the measured data at 77 and 300 GHz.

Figure 11 shows the increasing trend of specific attenuation versus frequency at three different temperatures. The measured data for the specific frequency and temperature are also shown in Figure 11 for comparison purposes. A higher increase was observed for higher temperature due to higher steam density at higher temperature.

For cases of strong flame and strong smoke, we could not theoretically calculate the attenuation of the signal since we do not know the parameters of the plasma and the soot particles. As can be seen from our experiment, radar performance in these cases was high since the attenuation of the signal was low.

## 6. Conclusions

During our experiments, we investigated the propagation of electromagnetic signals with frequencies of 300 and 79 GHz through fire to assess the possibility of using radars to obtain images in fire environments. The radar performance was evaluated under various real conditions, including fire with strong flame, dense smoke and water vapour.

In all scenarios considered by us, the radar provided reliable image acquisition of objects. The attenuation of a signal with a frequency of 300 GHz was, as expected, higher than with a frequency of 79 GHz. This is especially true for signal absorption in dense steam. Nevertheless, considering that the required visibility range usually does not exceed 10 m, we can conclude that a radar operating at a frequency of 300 GHz can be used for imaging in fire.

The analysis of the experimental results suggests good prospects for the use of Low THz radar in imaging devices for firefighters. Given the complexity and high cost of our full-scale experiments, it seems an important task to develop a model that describes the attenuation of signals in a fire. The results obtained in this study will serve as a basis for the verification of the model.

## Figures and Tables

**Table 1:** Measurement system characteristics.

Parameters	300 GHz	79 GHz
Frequency band	282–298 GHz	77–82 GHz
Wavelength	1 mm	3.8 mm
Bandwidth	16 GHz	5 GHz
Output Power	0 dBm	15 dBm
Azimuth Beamwidth	2.2° (−3 dB)	2.2° (−3 dB)
Elevation Beamwidth	15° (−3 dB)	15° (−3 dB)
Range resolution	9.4 mm	30 mm
Scan rate	0.83 Hz	0.83 Hz
Azimuthal step	0.25°	0.25°
Total azimuthal scan angle	40°	40°

**Table 2:** Radar Signal Attenuation (dB/m).

Environment	300 GHz	79 GHz
Heavy flame	0.09	0.02
Heavy smoke	0.13	0.09
Steam and particulate mix	0.88	0.45

## Data Availability

The data that support the findings of this study are available from the corresponding author, [AB], upon reasonable request.
